# A unifying perspective on neuromodulatory effects on signal transmission and plasticity in D1-dominant MSN neurons

**DOI:** 10.1186/1471-2202-14-S1-P172

**Published:** 2013-07-08

**Authors:** Simon M Vogt, Ulrich G Hofmann

**Affiliations:** 1 AG Neuroelectronic Systems, University Clinic Freiburg, 79108 Freiburg, Germany; 2 Institute for Signal Processing, University of Lübeck, 23562 Lübeck, Germany

## 

How could phasic variations in dopamine level affect the learning outcome of a spiking neural network? How may neuromodulation affect the network's instantaneous response to simultaneously arriving glutamatergic inputs? How may this depend on the brain regions involved?

In our spiking phenomenological model for signal transmission across the synapse and along the dendritic tree, we propose a new approach for the influence of dopamine-like neuromodulators on the ascribed aspects, which unifies diverging views on its role in (reinforcement) learning and (attentional) contrast.

We call into question the common practice of simulating dopaminergic influence on an STDP rule as a third factor, and instead show how an instantaneous effect of a dopamine-like neuromodulator on postsynaptic activity can also lead to reinforced learning outcomes.

As the phasic change of neuromodulator needs to be present during glutamatergic transmission in our model, we do not account for delayed reward as stated in the distal reward problem. Instead, we assume an involvement of hippocampus and cortical working memory for long delays of reward. However, as our transmission-based model does not interfere with the standard two-factor STDP rule, it may be freely combined with existing extensions to STDP if needed.
